# Outcome Quality of Inpatient and Day-Clinic Treatment in Child and Adolescent Psychiatry—A Naturalistic Study

**DOI:** 10.3390/children8121175

**Published:** 2021-12-11

**Authors:** Leonhard Thun-Hohenstein, Franka Weltjen, Beatrix Kunas, Roman Winkler, Corinna Fritz

**Affiliations:** 1 Paediatric and Adolescent Psychiatry, University Children’s Hospital, Paracelsus Medical University (PMU), 5020 Salzburg, Austria; f.weltjen@gmail.com (F.W.); beatrix.kunas@stud.sbg.ac.at (B.K.); c.fritz@salk.at (C.F.); 2 Ludwig Boltzmann Institut for Health Technology Assessment, Ludwig Boltzmann Gesellschaft, 1090 Vienna, Austria; roman.winkler@goeg.at; 3 Institute of Psychology, Paracelsus Private Medical University, 5020 Salzburg, Austria

**Keywords:** child and adolescent psychiatry, inpatient, day-clinic, outcome quality, treatment satisfaction, quality of life

## Abstract

Background: Child and adolescent psychiatry has only recently been established as a separate specialty and is practiced in different settings. The epidemiology of psychological problems in childhood is high and varied, thus qualitative work is essential. Assessment of outcome as part of quality management is central to assure the service of psychiatric care to be effective. Method: Over a three-year period consecutively admitted patients from inpatient and day-clinic treatment were prospectively evaluated. A total of 200 from 442 patients (m = 80, f = 120; age 15.1 ± 2.8 y) agreed to participate. Patients, caregivers, and therapists answered a range of questionnaires to provide a multi-personnel rating. Questionnaires used for outcome assessment were Child Behavior Checklist (CBCL) and Youth-Self-Report (YSR) (at admission, discharge, and 6 weeks after discharge) and the problem score of the Inventory of Quality of Life for children (ILK), treatment satisfaction, and process quality by the Questionnaire for Treatment Satisfaction (FBB, at discharge) and as real-life outcome control assessment of quality of life (ILK) was added (admission, discharge, and 6 wks after discharge). Results: There was a significant reduction in psychopathologicalsymptoms (CBCL, YSR) and in the problem score. Furthermore, there was a significant increase in quality of life. QoL score and YSR/CBCL scores returned to normal levels. Treatment satisfaction was high and so was satisfaction with process quality. Factors significantly influencing outcome were severity of disease and the relationship to the therapist. No differences were found for gender and setting. Conclusion: The quality management analysis revealed significant improvements of symptom load, a significant increase in QoL and a high treatment satisfaction. Furthermore, process quality was scored highly by parents and therapists.

## 1. Introduction

In Austria, child and adolescent psychiatry (CAP) has only recently become a separate medical specialty by Austrian federal law. Up to 2007, when law was passed [1], it used to be an additive special medical education, only accessible for pediatricians, neurologists, and psychiatrists. The prevalence of psychological impairment among children and adolescents affects over 13.0% of the age group worldwide, 17.0% in Germany, and up to 35.0% in Austria [2]. Both late creation of the specialty and the epidemiology necessitate an increased need for intervention and prevention [3]. Diagnostic and therapeutic services are provided in private and public practice, day-clinics, outpatient clinics, and hospitals of different levels. During the last ten years, CAP services in Austria have grown significantly, but not sufficiently to meet the need for treatment [4].

Thus, the topic of quality management in the treatment of those affected is of particular importance. Quality management generally refers to—as defined by Austrian federal definition (ÖNORM EN ISO 9000:2000)—coordinated activities for the management and direction of an organisation, which aim to improve the quality of the products produced or the service offered. Theoretically, hospitals in Austria are obliged to systematically implement quality management, but until now there has been very little progress, as there are no officially defined benchmarks for CAP implementation. Bickman et al. [5] recommend the following topics to be included in quality strategies in child mental health: the severity and acuity of the child’s symptoms; the child’s functional impairment; the child’s functional strengths; family functioning; the quality of family life; consumer satisfaction; the goals of treatment; the modality, strategy, and tactics of treatment; readiness for change; the quality of the therapeutic alliance and adherence to treatment. Usually, three areas are distinguished in quality management: structural quality, process quality and outcome quality [6].

Between 2004 and 2009 a new university department for child and adolescent psychiatry was developed in Salzburg. As part of a year-long organisational development process, a new departmental structure was developed as well as a basic treatment concept for inpatients and day-clinic patients [7]. For this, the structure of service and various working processes was defined, and the results were documented in an organisational manual. This is available to all employees via an online platform (Medikit https://medikit.net/de/).

The treatment concept is based on a systemic psychotherapeutic concept and aims to establish optimal cooperation conditions between the multiprofessional team, the patients and their families or guardians [8,9,10]. The systemic understanding of treatment places the patient in relationship to his network of relationships and understands psychological impairments as interactional disorders in the system [11]. The embedding of patients in relationship systems has a significant influence on the success of treatment [12]. Therapists and other support structures such as the care offered at the clinic act as new actors in the system and can activate resources [9].

All processes were designed according to the topics of the above-mentioned quality aspects, especially participation [13], solution orientation and resource orientation [8] and help for self-help. A distinction is made between different types of stay, each of which includes a specific and standardized procedure: acute stay (risis intervention), orientation stay (multimodal diagnostics and clearing) and project stay (psychotherapeutic treatment stay). These forms also differ in terms of motivation: in contrast to acute and orientation stays, project stays are planned electively and voluntarily with generally high motivation. In addition, depending on the diagnosis, disease-specific concepts (e.g., eating disorder treatment, etc.) are applied at the department–relying on the systemic concept.

After the development of the conceptual part and its implementation, an external assessment of employee rating (1–5; *n* = 43/55) concerning communication/cooperation, information/participation and organisation showed a high level of satisfaction (communication mean 1.85 ± 0.77, information 1.68 ± 0.68 and organisation 1.97 ± 0.7; personal data); additionally, the clinical impression of the implementation was of a very high standard. This gave rise to the idea of also investigating the outcome quality of the new therapeutic structures and processes.

Evaluation of outcome quality under most naturalistic conditions is described to be the possibly best case to ensure practical generalizability of results [14]. Furthermore, since this is no biological or physiological study [15] and to maintain control of our organisational and conceptual implementations, we decided to follow the concept of a naturalistic study. There is a lack of such studies, especially in children and adolescent psychiatry. Solid evaluations following the underlying systemic theory should be “multi-perspective”, i.e., the various health professionals, parents, or primary caregivers and the children and adolescents themselves, should be included in the evaluation.

Foundations in the field of evaluation of child and adolescent psychiatry have been laid by the works of Remschmidt and Mattejat [16,17]. These authors introduced the Marburg evaluation project (Marburger System zur Qualitätssicherung und Therapieevaluation, MARSYS; [17]) which systematically investigated the success of the treatment in a local child and adolescent psychiatry hospital under naturalistic conditions. This work was used as a basis for the present evaluation project’s structure and intention. Although Remschmidt and Mattejat’s study is one of the pioneering works in Germany, there are only two Austrian studies on inpatient treatment outcomes in Austrian CAP departments [18,19].

The primary focus of assessment of outcome quality lies on the success of treatment. The comparison of a pre–post measurement of the extent of symptoms provides the central measure for evaluating treatment success. Evaluative studies in child and adolescent psychiatry consistently show a positive change in symptoms regardless of the disorder [19,20,21,22,23] as well as in a disorder-specific context [16]. The quality of the relationship between the patients and the therapist contributes significantly to the success of the treatment [24].

In addition to this primary parameter, recent research has been increasingly focused on the analysis of additional factors affecting treatment conditions in evaluations [16,20]. Closely associated with the success of treatment is treatment satisfaction within all participants in the treatment process. In the literature, the two parameters are regarded as the same construct (i.e., treatment success is determined by the treatment satisfaction; [22]) and as parallel constructs that correlate positively with each other [16,21]. In general, at least a moderate treatment satisfaction is achieved at the end of a successful treatment [17,25].

Treatment success and treatment satisfaction do not predict whether the children are going to do well in real life. It is important to look at the clinical significance of the results and, thus, the aspect of quality of life of patients has recently gained importance in medical evaluation research [26]. This is crucial, since, simply considering the change in clinical symptoms does not suffice to make valid statements on the improvement of function for the patients [27]. The additional recording of quality of life as a separate construct can, therefore, provide valuable additional information [28]. Reduced quality of life turns out to be a systematic feature of mentally impaired children and adolescents [29,30]. Consequently, this is increasingly regarded as a recommendation for therapeutic practice [31,32].

It has been shown that successful treatment is accompanied by an increase in the quality of life [16,28,33,34], as well as an increased accordance between the perspectives of all participants in the treatment process. [18,19,30]. Different perspectives can also provide exclusive information about the quality of life of patients [35].

Thus, for evaluating the results of the organisational project and the therapeutic concept a naturalistic study was designed, investigating treatment success, treatment satisfaction, and quality of life. It was assumed that the therapeutic concept provides significant reduction of symptoms with clinical relevance, accompanied by high treatment satisfaction and significant improvement of the quality of life.

In addition, the influences of gender and age on treatment success and treatment satisfaction were examined as various framework conditions of treatment: these included differences in the form of stay and the influence on the therapeutic relationship.

## 2. Materials and Methods

The study period lasted from April 2011 to January 2014 and was approved by the Salzburg Ethics Commission under E-1195 (28 April 2010). Within this period, at the time of admission, all patients were asked if they were willing to participate in the study. In the event of consent, participants and custodial providers were asked to sign a written declaration of consent (EVE). In the event of rejection, there were no disadvantages for the patients at any time during treatment. The sample also included the primary caregivers of the children and adolescents, as well as the treating physicians, psychologists or psychotherapists, educators, and social workers. Patients under the age of six years and patients displaying acute suicidality, psychosis, or cognitive impairment were excluded from the study. In the case of insufficient knowledge of German, interpreters were consulted. In the primary data collection, a total of four measurement time points were chosen: admission to the clinic (T1), discharge (T2), catamnesis six weeks after discharge (T3) and another catamnestic survey 18 months after discharge from the clinic (T4). The survey took place in a specially provided room, accompanied by study assistants. In the present work the measurement at timepoint T4 is not considered.

### 2.1. Measures

The data were collected using quantitative questionnaires. Treatment success, treatment satisfaction, and quality of life were measured multi-perspectively with different instruments at three time points: admission, discharge, and 6 weeks after discharge. Questionnaires were filled out digitally; patients were accompanied by a psychologist.

### 2.2. Instruments

*Treatment success*—as defined by a significant reduction of symptoms i.e., psychopathology, between T1, T2, and T3—was measured by Youth Self-Report (YSR) and Child Behavior Checklist (CBCL), using the Total Problem Scale, Internalizing and Externalizing scale [36].

*Treatment satisfaction* was measured by the Questionnaire for Treatment Satisfaction (FBB; Mattejat & Remschmidt, [37]), providing rating of treatment satisfaction by patients, parents/caregivers, and therapists. Statements are rated using a 5-point Likert scale, ranging from 0 (don’t agree at all) to 4 (agree completely). This questionnaire also can be used as a quality assessment instrument, dividing the results into outcome (items 1,3,18,20 and 6) and process quality (all other items). Subscores for caregivers and patients were calculated for outcome quality: personal success and family success and for process quality: relationship to therapist and framework conditions. The latter were only rated by caregivers, as suggested by the manual. Internal consistency (Cronbach’s alpha) is reported to be 0.88.

*Quality of life* (QoL) was measured by the Inventory for quality of life (ILK; Mattejat & Remschmidt, [38]) in children and adolescents and parents/caregivers. For analysis, the total score for quality of life and the problem score, assessing load of the disease and the treatment, were employed. Internal consistency (Cronbach´s alpha) for the total score is reported to be 0.55–0.76. Normative data are provided for healthy and mentally ill children and adolescents.

### 2.3. Statistical Analysis

Both correlation and difference hypotheses were formulated and evaluated with the program IBM SPSS Statistics 24 (IBM, Armonk, NY, USA) and R (version 4.0.1, R Core Team, Vienna, Austria) for Windows. If the requirements for the use of parametric methods were not met, their non-parametric equivalents were used. For the difference hypotheses in independent design, the Mann–Whitney U test was used for the comparison of two groups and the Kruskal–Wallis test for the comparison of more groups. Pairwise comparisons were performed in the Kruskal–Wallis test using the non-parametric post hoc test according to Dunn (Bonferroni correction). For the difference hypotheses in the dependent design, the t-test for dependent samples or the Wilcoxon signed-rank test was chosen for two samples, as well as the Friedman test for more than two groups. The correlation hypotheses were analyzed using spearman’s rank correlation coefficient. For statistical description, absolute and relative frequency data, mean values, and standard deviations were used. If possible, 95% confidence intervals and effect sizes were specified. For the description of the effect sizes, the measure of Cohen’s d was chosen for the mean value differences, and the correlation coefficient r was employed to evaluate the differences between medians and in the correlation calculations. The hypothesis tests were subject to two-sided calculations with a significance level *p* of *p* = 0.05 (*), *p* = 0.01 (**) and *p* = 0.001 (***). For the analysis of the hypotheses, in the case of missing data, the list-by-list case exclusion was chosen.

#### Sample Characteristics

**Figure 1:**
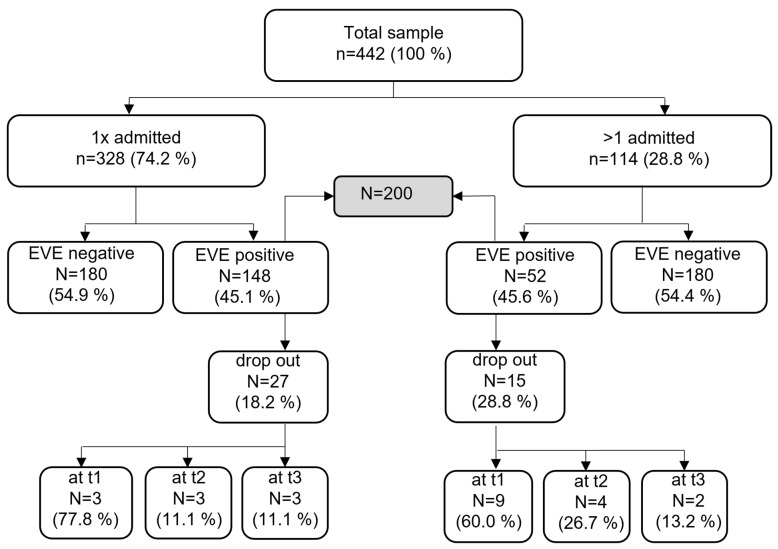
Consort diagram of study sample; EVE acceptance signature.

The analyses included data from 442 patients treated during the aforementioned study period. Of this total, 328 were hospitalized once and 114 multiple times throughout the study period. Only the first hospitalization was included in the calculations. The consent for participation was given by *n* = 200, 148 of which were admitted once and 52 of which were admitted several times. Figure 1 illustrates the sampling process graphically. The sample size varies depending on the questionnaire and time of testing. At test time T1, data from 170 patients (85.0%) were available, while 163 data sets (81.5%) were available for T2 and 158 data sets (79.0%) for T3.

In the sample with positive consent (*n* = 200), there were 80 male (40.0%) and 120 female (60.0%) patients. The average age of patients was 15.14 years (SD = 2.83, [5,21]; female: M = 15.45, SD = 2.08, [7,21]; male: M = 13.49, SD = 3.42, [5,17]), with most patients in the age group between 14 and 17 years (*n* = 135, 67.5%). Diagnosis was classified at the end of hospitalization in 197 patients: anxiety disorders (F4; *n* = 74, 37.0%), eating disorders (F5; *n* = 33, 16.5%) and behavioral and emotional disorders (F9; *n* = 50.0, 25.0%). The average duration of treatment was 57.34 days (SD = 58.16, [1, 305]), with most patients (*n* = 94; 47.0%) being treated for more than 41 days. As a form of stay, 114 patients were in crisis stay (57.0%), while 67 patients were in orientation stay (33.5%) and 19 patients were in project stay (9.5%).

Patients with positive and negative EVE differed significantly regarding form of stay and form of admission (planned or unplanned). Patients with positive EVE were more likely to be admitted on a planned basis (U = 15,842, *p* < 0.001) and patients with negative EVE were more likely to be in crisis stay (U = 17,043, *p* <.001). Further descriptive information on the sample and a comparison of the characteristics of the subjects included as opposed to the not included are given in the Appendix A.

## 3. Results

Overview of all results is summarised in Table 1.

### 3.1. Treatment Success—Reduction of Psychopathology

The clinical symptoms rated by the children and adolescents showed a highly significant decrease between the times T1, T2, and T3 (χ^2^ = 84.8, *p* < 0.001) indicating treatment success. There was no difference between boys and girls (*U* = 3512.5, *z* = 0.57, *p* = 0.571, *r* = 0.04). Comparison of age groups (≤6; 6.1–14; 14–18; >18) showed no significant differences (2708; df = 3; *p* = *0*.439).

The effect sizes are in the high range (T1–T2: r = 0.66; T1–T3: r = 0.88), while the effect size between T2 and T3 lies in the middle range (r = 0.21). This decrease also applies to the reported internalizing and externalizing symptoms (internalizing: χ^2^ = 76.85, *p* < 0.001; externalizing: χ^2^ = 36.14, *p* < 0.001): here, too, the effect sizes can be found in a similar spectrum (internalizing, T1–T2: r = 0.64; T1–T3: r = 0.81; T2–T3: r = 0.17; externalizing, T1–T2: r = 0.37; T1–T3: r = 0.57; T2–T3: r = 0.21). Assessment of the symptom change rated by the main caregivers showed a comparable picture of a significant decrease in clinical symptoms (χ^2^ = 80.07, *p* <.001). This effect applies also to both the internalizing and externalizing symptoms (internalizing: χ^2^ = 57.91, *p* < 0.001; externalizing: χ^2^ = 56.38, *p* < 0.001). The results are summarised in Table 2.

### 3.2. Treatment Satisfaction

On average patients showed positive treatment satisfaction (M = 2.94, SD = 0.63), a result that is significantly different from zero (t = 60.19, *p* < 0.001) and lies above the normative data (clinical sample mean 2.57 ± 1.31). There were no significant differences between treatment satisfaction of male and female patients (U = 3301.50, z = −0.12, *p* = 0.903). Treatment satisfaction also turned out to be equivalent in the different age groups (χ^2^ = 4.34, *p* = 0.227).

With regard to the form of hospitalization, there was no significant effect (H(15) = 5.64, *p* = 0.060, η^2^ = 0.02, d = 0.39). A significant association was found between the severity of clinical symptoms at T1 and treatment satisfaction at T2. This effect applies equally to the age groups of children and adolescents, i.e., the higher the clinical symptoms were, the lower the treatment satisfaction of both children and adolescents was (children: rS = −0.56, 95% BCa CI [−0.78, −0.23], *p* < 0.01; Adolescents: rS = −0.27, 95% BCa CI [−0.42, −0.11], *p* < 0.01).

In relation to the quality assessment, the results of the FBB analysis at T2 are shown in Table 3, showing a high satisfaction of caregivers and patients concerning the different quality measures. Framework quality correlates significantly with outcome quality rated by patients (rS = 0.56, 95% BCa CI [0.43, 0.67], *p* < 0.001) and caregivers (rS = 0.25, 95% BCa CI [0.06, 0.43], *p* < 0.05.), i.e., the better the framework the higher the satisfaction with treatment.

### 3.3. Quality of Life

At T1 quality-of-life score was lower as the norm sample and significant improvements in quality of life were observed over the treatment period (see Table 2). At T3 the mean QoL score was above the norm population (19.25) for parents (19.47 ± 4.5), children (21.0 ± 5.6) and adolescents (21.0 ± 4.25). Large effects (Cohen’s d ≥ 0.8) were found in terms of the overall assessment of quality of life (adolescents: z = 5.51, *p* < 0.001, d = 1.22; Main caregivers: z = 6.61, *p* < 0.001, d = 1.81) and mental health assessment (adolescents: z = 6.91, *p* < 0.001, d = 1.71; main reference persons: z = 5.71, *p* < 0.001, d = 1.42). Additionally, a significant reduction in the problem score (adolescents: z = 6.88, *p* < 0.001, d = 1.71; Main caregivers: z = 5.09, *p* = < 0.001, d = 1.21) and a significant increase in the quality-of-life score 0–28 (adolescents: z = −7.72, *p* < 0.001, d = −2.13; Main reference persons: z = −6.43, *p* < 0.001, d = −1.72) illustrates an improvement in quality of life from the time T1 to T3. Furthermore, there was no significant difference between boys and girls (U = 1275.5; *p* = 0.775).

In the sample of children admitted to the UK-KJP (*n* = 24), a significant improvement in quality of life was found as well. The quality-of-life score at T2 is reported significantly higher than at T1 (z = −2.96, *p* = 0.009, d = −1.52), although this increase in quality of life is no longer detectable at T3 (z = −1.80, *p* = 0.214, d = −0.79); a result which may occur due to the small sample size.

## 4. Discussion

This study measures outcome quality within a naturalistic design at a department for child and adolescent psychiatry. Results show a significant reduction of psychopathology from admission to discharge and until six weeks after discharge. Effect sizes are high during the time of treatment and smaller, yet still significant, after discharge with high effect sizes also for general psychopathology as well as internalizing and externalizing symptoms. This means, the therapeutic setting provided leads to a significant symptom reduction. Symptom reduction is the first and main goal of treatment and, thus, the main outcome criteria for quality assessment [39]. In comparison to the two other Austrian studies [18,19] our results are aligned with previous findings. One of the departments evaluated by the older Austrian studies is also grounded on systemic therapy theory, the second is grounded on psychoanalytical theory. All three studies are naturalistic studies without a control group, without randomization and crossover, thus, mirroring the reality of daily treatment (treatment as usual) all over the world. It would be interesting to analyze the similarities of those departments in treatment provision, structure, and processes, to gain causal information about what “really helps”. One principle of evidence-based medicine is to reach informed consent or shared decision-making, which this treatment process provides on a high level [13]. The other reason to perform a naturalistic study was the reality of treatment provision to kids of different ages, sex, with various different diseases and at different stages of disease, criteria which no known EBM protocol could provide. The therapeutic concept used in this study was developed to create a basic treatment situation, which is the grounding basis for an individual’s recovery.

In all three Austrian studies treatment is successful in the sense of symptom reduction and clinical relevance, thus, arguably reaching at least a partial recovery—a results which has been documented by comparable international studies as well [20,21,22,23]. Recovery is defined as a “profound personal … process to change attitudes, values, feelings, abilities, and roles to achieve a satisfactory, hopeful and productive way of life with the possible limitations of illness” [40]. Certainly, our study can only demonstrate recovery in the sense of symptom reduction and increased quality of life, but any recovery is important for lifelong prognosis of mental disorders. As a longer duration of mental health problems in adolescence is the main prognostic factor, it is essential to reduce symptom load and increase self-efficacy and quality of life [41]. Therefore, it is crucial to achieve a clinically relevant reduction. In our study, this is exemplified by reaching the normative symptom level of the CBCL scales and by the significant increase of the QoL score. Furthermore, the problem score of the QoL questionnaire also decreased significantly, providing even more information about the clinical relevance of the reported outcome quality.

From a quality management perspective treatment success—defined as satisfaction with treatment and with process—is the most important parameter. In our study, treatment satisfaction is high for caregivers, less but also very positive for patients and high for therapists. Looking at the subscores outcome quality is also in the high range and so are framework conditions and relationship to therapist. Both factors of process quality proved to be significantly associated with high satisfaction with treatment. In a recent qualitative survey Schneidtinger and Haslinger-Baumann [42] describe a model of recovery with 3 main facilitating factors. The basic level consists of supporting therapies, help with daily structures and the ward as a safe place. The next level is provided by peers´ understanding, community, and friendship, the third level by family factors as connectedness and parental support. As parents and patients rate the framework conditions satisfying the concept of department delivers the basic level of the recovery model. As parents are rating the improvement in family function equally positively as their children, effects on the third level can be assumed.

For future quality management in child and adolescent psychiatry there are several conclusions to draw. First, outcome measurement as performed in this study is useful and should be used in a continuous quality improvement concept [5,43] and performed as a measurement feedback system [44]. Reduction of symptoms and treatment satisfaction could be used as benchmarks, quality-of-life measures, and reduction of problem scores added for control of clinical relevance. However, as “recovery” would be the central goal of a profound quality management the quantitative assessment should be enriched by detailed analysis of, e.g., the severity and acuity of the child’s symptoms; the child’s functional impairment; the child’s functional strengths; family functioning. Furthermore, assessment of the goals of treatment; the modality, strategy, and tactics of treatment; readiness for change; the quality of the therapeutic alliance and adherence to treatment should be added to the quantitative assessment of “recovery” [43].

Kelly [44] recommends multidimensional monitoring in three modules: a baseline follow-up module which assesses the child’s and family’s mental health status starting the entry to the system and lasting throughout treatment; a concurrent module which obtains information throughout the course of treatment; and a background module which assesses factors in the child’s and family’s background that moderates the course of treatment.

Furthermore, regular assessments of the employees’ view of the structures and processes as well as their implementation—together with the measures described above—would give a 360-degree view of the therapeutic process a team provides. The results should be regularly assessed and reported back to the providing team for control, reflection, and supervision.

### Limitations

In the sense of the strict rules of evidence-based medicine with the necessity of double blinded, randomized crossover studies the naturalistic design is the main limitation of this study. Nevertheless, critics of this strict interpretation of evidence-based research suggest also using alternative methods for gaining evidence [15]. The naturalistic approach is thought to evaluate concepts in the real situation, closely monitoring the real processes of the applied treatment service.

Moreover, participation of patients and caregivers was not complete, thus this may have caused a bias, although we checked the anamnestic details and found no difference between the participants and the non-participants concerning age, gender and several psychosocial items (see also Table A1).

## 5. Conclusions

Our study demonstrates strong therapeutic effects of a stringently organised and reflected multiprofessional treatment approach. Systemic theory gave rise to the values, vision, and mission of the project; it provided the basis for developing the respective structures and processes. Symptom reduction and treatment satisfaction is shown to be high, effect sizes are strong, and the clinical relevance—as measured by quality of life at discharge—is comparable to healthy children and adolescents.

## Figures and Tables

**Table 1:** Overview of sample sizes, means, and *SD* for all scales and participants.

	T1	T2	T3
FBB			
Therapists *n* (%)		131 (68.23)	
Therapists total score *M (SD)*		2.79 (0.51)	
Patients *n* (%)		128 (66.67)	
Patients total score *M (SD)*		2.98 (0.65)	
Main caregivers *n* (%)		99 (51.6)	
Main caregivers total score *M (SD)*		3.28 (0.57)	
CBCL *n* (%)	113 (54.5)	105 (52.5)	96 (48)
Externalizing problems *M (SD)*	61.89 (10.67)	58.08 (9.74)	57.13 (10.51)
Internalizing problems *M (SD)*	69.73 (8.92)	64.50 (9.27)	61.68 (11.44)
Total problems *M (SD)*	69.67 (8.13)	64.23 (8.45)	61.72 (10.83)
YSR *n* (%)	164 (82)	122 (61)	104 (52)
Externalizing problems *M (SD)*	58.05 (9.86)	54.12 (9.89)	53.02 (9.87)
Internalizing problems *M (SD)*	65.98 (9.88)	60.24 (11.26)	57.47 (11.15)
Total problems *M (SD)*	65.48 (9.06)	59.41 (10.89)	57.31 (10.26)
ILK			
Main caregivers *n* (%)	116 (58)	105 (52.5)	96 (48)
Problem score PR_0–7_ *M (SD)*	3.73 (1.67)	2.47 (1.86)	2.55 (2.07)
Quality-of-life score LQ_0–28_ *M (SD)*	16.41 (4.16)	19.36 (3.90)	19.47 (4.50)
Adolescents *n* (%)	148 (74)	111 (55.5)	94 (47)
Problem score PR_0–7_ *M (SD)*	3.34 (1.59)	2.08 (1.97)	1.96 (1.86)
Quality-of-life score LQ_0–28_ *M (SD)*	17.55 (4.01)	20.69 (4.48)	21.01 (4.25)
Children *n* (%)	28 (14)	26 (13)	23 (11.5)
Problem score PR_0–7_ *M (SD)*	2.75 (1.69)	2.15 (1.49)	2.3 (2.12)
Quality-of-life score LQ_0–28_ *M (SD)*	19.18 (4.36)	21.58 (3.84)	21.00 (5.55)

Abbr. FBB for Treatment Satisfaction; CBCL Child behavior Check List, YSR Youth self report; ILK Inventory for Quality of Life.

**Table 2:** Treatment success and quality of life over time and results of significance tests.

Measuring Instrument	Perspective	T1 Median	T2 Median	T3 Median	χ^2^ Test Statistics
YSR	Patients’ self-report				
	YSR-INT (*n* = 99)	21	13	10	76.85 ***
	YSR-EXT (*n* = 99)	13	10	9	36.14 ***
	YSR-GES (*n* = 99)	58	40	35	84.80 ***
CBCL	Main caregivers’ rating				
	CBCL-INT (*n* = 87)	20	13	11.5	57.91 ***
	CBCL-EXT (*n* = 86)	13.5	10	8.5	56.38 ***
	CBCL-GES (*n* = 86)	57.5	37	34	80.07 ***
ILK	Children’s self-report (*n* = 24)				
	ILK-PR_0–7_	3.00	2.00	2.00	2.03
	ILK-LQ_0–28_	19.00	21.00	22.00	10.17 **
	Adolescents’ self-report (*n* = 112)				
	ILK-PR_0–7_	4.00	2.00	2.00	78.42 ***
	ILK-LQ_0–28_	18.00	21.00	21.00	85.19 ***
	Main caregivers’ rating (*n* = 97)				
	ILK-PR_0–7_	4.00	3.00	2.00	48.47 ***
	ILK-LQ_0–28_	16.00	20.00	20.00	63.46 ***

*Note. n* = quantity. The table refers to the initial admissions of patients admitted to UK-KJP with positive informed consent. Means (M) and standard deviations (SD) of the CBCL, YSR, and FBB procedures were calculated using T values. The scales of the ILK have specific limits, *see indices*.

**Table 3:** Quality measures according FBB: percentage of categories 3 (mainly correct) and 4 (precisely correct) as well as the mean of the means of all ratings of patients (*n* = 111) and caregivers (*n* = 118).

Quality Measure	Submeasure	Caregivers		Patients	
		Mean %	Mean * Mean	Mean %	Mean * Mean
Outcome Quality	personal success	73.3 ± 13.7	3.2 ± 0.4	74.9 ± 5.0	3.0 ± 0.2
	family success	92.1	3.6 ± 0.7	53.3	2.6 ± 1.3
Process Quality	relation to therapist	83.8 ± 14.2	3.3 ± 0.4	85.4 ± 5.6	3.3 ± 0.1
	framework conditions	81.5 ± 5.8	3.1 ± 0.2		

FBB Questionnaire for treatment satisfaction. * Scale: 0 no success, 1 rather successless, 2 partially successful 3 mainly successful, 4 fully successful; shaded areas were not included in questionnaire for patients.

## Data Availability

The data generated and analysed during the present study are not publicly available due to ethical restrictions but are available from the corresponding author upon reasonable request.

## Appendix A

**Table A1:** Sample characteristics and comparison of participants and non-participants.

Sample Characteristics		Total Sample	Sample with Positive EVE	Sample with Negative EVE
		*n* (%)	*n* (%)	*n* (%)
(Total) Age		442 (100)	200 (100)	242 (100)
≤6 years (Children ^2^)		5 (1.1)	4 (2)	1 (0.4)
≥7 to ≤13 years (underage minors ^2^)		105 (23.8)	45 (22.5)	60 (24.8)
≥14 to ≤17 years (minors of age ^3^)		311 (70.4)	135 (67.5)	176 (72.7)
≥18 years (Adults ^3^)		21 (4.8)	16 (8)	5 (2.1)
Sex				
female		250 (56.6)	120 (60)	130 (53.7)
male		192 (43.4)	80 (40)	112 (46.3)
Diagnosis ^1^ at T2		-	197 (98.5)	0 (0)
F3			23 (11.5)	
F4			74 (37.0)	
F5			33 (16.5)	
F9			50 (25.0)	
Other (F1, F2, F6, F8)			17 (8.5)	
Missing			3 (1.5)	
Hospitalization form				
planned		133 (30.1)	98 (49)	35 (14.5)
unplanned		309 (69.9)	102 (51)	207 (85.5)
Type of stay				
Crisis stay		322 (72.9)	114 (57)	208 (86)
Orientation stay		97 (21.9)	67 (33.5)	30 (12.4)
Project stay		23 (5.2)	19 (9.5)	4 (1.7)
Department				
Day-clinical stay		63 (14.3)	46 (23)	17 (7)
In-patient stay		379 (85.7)	154 (77)	225 (93)
Duration of stay				
≤1 day		96 (21.7)	5 (2.5)	91 (37.6)
≥2 to ≤7 days		132 (29.9)	33 (16.5)	99 (40.9)
≥8 to ≤41 days		105 (23.8)	68 (34)	37 (15.3)
≥42 days		109 (24.7)	94 (47)	15 (6.2)
Legal basis of inpatient admission ^1^		-	192 (100)	0 (0)
voluntary			152 (79.2)	
according to § 8 UBG			25 (13)	
according to § 9 UBG			1 (0.5)	
by court order			1 (0.5)	
other			13 (6.8)	
Legal duty of custody ^1^		-	192 (100)	0 (0)
father			9 (4.7)	
mother			69 (35.9)	
both parents			97 (50.5)	
youth welfare			7 (3.6)	
other			10 (5.2)	
Parents’ school-leaving qualification ^1^		-	368 (100)	0 (0)
No school-leaving qualification	Mother		2 (0.5)	
	Father		0 (0)	
Special education school	Mother		1 (0.3)	
	Father		2 (0.5)	
Secondary school	Mother		41 (11.1)	
	Father		19 (5.2)	
Apprenticeship	Mother		63 (17.1)	
	Father		71 (19.3)	
Abitur/A-Level/vocational baccalaureate diploma	Mother		38 (10.3)	
	Father		26 (7.1)	
University degree	Mother		22 (6)	
	Father		26 (7.1)	
Unknown	Mother		17 (4.6)	
	Father		40 (10.9)	

*Note*: *n* = quantity. ^1^ Data were taken from baseline documentation (BADO). Therefore, no data are available for the sample with negative EVE. ^2^ Designated as children in the present study. ^3^ Designated as adolescents in the present study.

## References

[1] Ärzteausbildungsordnung (Medical Doctors’ Education Regulation) Regulation of the Federal Minister of Health on Training as a General Practitioner and as a Specialist; 2015,) StF: BGBl. II Nr. 147/2015 §15(1)13. https://www.ris.bka.gv.at/GeltendeFassung.wxe?Abfrage=Bundesnormen&Gesetzesnummer=20009186.

[2] Philipp J., Zeiler M., Waldherr K., Nitsch M., Dür W., Karwautz A., Wagner G. (2014). The Mental Health in Austrian Teenagers (MHAT)-Study: Preliminary results from a pilot study. Neuropsychiatrie.

[3] Beck N., Warnke A. (2009). Youth welfare needs after inpatient child and adolescent psychiatric treatment. Zschr. Kinder Und Jugen Psychiatr. Psychother..

[4] Fliedl R., Ecker B., Karwautz A. (2020). Child and adolescent psychiatric care 2019 in Austria—Steps of care, current state and lookout. Neuropsychiatrie.

[5] Bickman L., Noser K. (1999). Meeting the challenges in the delivery of child and adolescent mental health services in the next millennium: The continuous quality improvement approach. Appl. Prev. Psychol..

[6] Kunkel S., Rosenquist U., Westerling R. (2007). The structure of quality systems is important to the process and outcome, an empirical study of 386 hospital departments in Sweden. BMC Health Serv. Res..

[7] Thun-Hohenstein L., Kerbl R., Thun-Hohenstein L., Vavrik K., Waldhauser F. (2008). Care situation of psychologically conspicuous and sick children and adolescents. Kindermedizin–Werte Versus Ökonomie.

[8] de Shazer S., Berg I.K., Lipchik E., Nunnally E., Molnar A., Gingerich W., Weiner-Davis M. (1986). Brief Therapy: Focused Solution Development. Fam. Process..

[9] Schweitzer J., Beher S., Sydow K., Retzlaff R. (2007). Systemic therapy / family therapy. Psychotherapeutenjournal.

[10] Walter G. (1998). Inpatient treatment as transition. Systeme.

[11] Working Group Systemic Therapy (2009). Statement on the Theory and Practice of Systemic Therapy. https://www.dgsf.org/themen/berufspolitik/berufspolitik-bis2005/agst_stellungn_wiss_beirat.html.

[12] Rotthaus W., Wirsching M., Scheib P. (2002). Systemic child and adolescent psychiatry and psychotherapy. Paar-Und Familientherapie.

[13] Thun-Hohenstein L. (2014). Participation of children and adolescents in child and adolescent psychiatry. Paediatr. Paedol..

[14] Heekerens H.P. (2005). From the laboratory to the field. Psychotherapeut.

[15] Falissard B. (2015). How should we evaluate non-pharmacological treatments in child and adolescent psychiatry. Eur. Child Adolesc. Psychiatry.

[16] Mattejat F., Trosse M., John K., Bachmann M., Remschmidt H. (2006). Model Research Project on the Quality of Outpatient Child and Adolescent Psychiatric Treatments, Final Report.

[17] Mattejat F., Remschmidt H. (2006). The assessment of therapy outcome in child and adolescent psychiatry under naturalistic conditions—Conception and implementation of the Marburg System of Quality Assurance and Therapy Evaluation. Z. Kinder Jugendpsychiatrie Psychother..

[18] Katzenschläger P., Fliedl R., Popow C., Kundi M. (2018). Quality of life and satisfaction with inpatient treatment in adolescents with psychiatric disorders. Neuropsychiatrie.

[19] Reinelt P.D. (2010). Treatment Assessment and Quality of Life in Child and Adolescent Psychiatry Inventories, Progress and Correlation Analyses. Bachelor’ Thesis.

[20] Bachmann M., Bachmann C.J., John K., Heinzel-Gutenbrunner M., Remschmidt H., Mattejat F. (2010). The effectiveness of child and adolescent psychiatric treatments in a naturalistic outpatient setting. World Psychiatry.

[21] Bredel S., Brunner R., Haffner J., Resch F. (2004). Treatment success, treatment experience and treatment satisfaction from the point of view of patients, parents and therapists—results of an evaluative study from inpatient child and adolescent psychiatry. Prax. Kinder Jugendpsychiatrie Psychol..

[22] Fleischhaker C., Bock K., Hennighausen K., Horwath D., Kuhn-Hennighausen C., Rauh R., Wewetzer G., Drömann S., Schulz E. (2008). 20-year catamnesis of the child and adolescent psychiatric and psychosomatic clinic Haus Vogt. Z. Kinder Jugendpsychiatrie Psychother..

[23] Gutknecht H. (2005). Patients’ evaluation of day-to-day clinical treatment–aspects of treatment experiences and changes experienced. Psychiatr. Prax..

[24] Cropp C., Streeck-Fischer A., Jaeger U., Masuhr O., Schröder A., Leichsenring F. (2008). The relationship between treatment experience and treatment success in inpatient psychotherapy with children and adolescents. Z. Kinder Jugendpsychiatrie Psychother..

[25] Kaplan S., Busner J., Chibnall J., Kang G. (2001). Consumer satisfaction at a child and adolescent state psychiatric hospital. Psychiatr. Serv..

[26] Renneberg B., Lippke S., Renneberg B., Hammelstein P. (2006). Quality of life. Gesundheitspsychologie.

[27] Reinecke M.A., Shirk S.R., Gabbard G.O., Beck J.S., Holmes J. (2005). Psychotherapy with adolescents. Oxford Textbook of Psychotherapy.

[28] Jozefiak T., Larsson B., Wichstrøm L., Wallander J., Mattejat F. (2010). Quality of Life as reported by children and parents: A comparison between students and child psychiatric outpatients. Health Qual. Life Outcomes.

[29] Balazs J., Miklosi M., Halasz J., Horváth L.O., Szentiványi D., Vida P. (2018). Suicidal Risk, Psychopathology, and Quality of Life in a Clinical Population of Adolescents. Front. Psychiatry.

[30] Bastiaansen D., Koot H.M., Ferdinand R.F. (2005). Determinants of quality of life in children with psychiatric disorders. Qual. Life Res..

[31] Baumgarten F., Cohrdes C., Schienkiewitz A., Thamm R., Meyrose A.K., Ravens-Sieberer U. (2019). Health-related quality of life and its relation to chronic diseases and mental health problems among children and adolescents: Results from KiGGS Wave 2. Bundesgesundheitsblatt Gesundh. Gesundh..

[32] Mattejat F., Simon B., König U., Quaschner K., Barchewitz C., Felbel DKatzenski B. (2003). Quality of life in mentally ill children and adolescents: Results of the first multicenter study with the inventory for recording the quality of life in children and adolescents (ILK). J. Child Adolesc. Psychiatry Psychother..

[33] Flechtner H., Möller K., Kranendonk S., Luther S., Lehmkuhl G. (2002). On the subjective quality of life of children and adolescents with mental disorders: Development and validation of a new survey instrument. Prax. Kinderpsychol. Kinderpsychiatr..

[34] Ravens-Sieberer U., Erhart M., Wille N., Bullinger M., BELLA Study Group (2008). Health-related quality of life in children and adolescents in Germany: Results of the BELLA study. Europ. Child Adolesc. Psychiatr..

[35] Kamp-Becker I., Schröder J., Muehlan H., Remschmidt H., Becker K., Bachmann C.J. (2011). Health-related quality of life in children and adolescents with autism spectrum disorder. Z. Kinder Jugendpsychiatr Psychoth..

[36] Döpfner M., Plück J., Kinnen C. (2014). German School-Age Forms of the Child Behavior Checklist by Thomas, M. Achenbach: Parent Questionnaire on Child and Adolescent Behavior (CBCL/6-18R), Teacher Questionnaire on Child and Adolescent Behavior (TRF/6-18R), Questionnaire for Adolescents (YSR/11-18R].

[37] Mattejat F., Remschmidt H. (1998). Questionnaires for Treatment Satisfaction.

[38] Mattejat F., Remschmidt H. (2006). Inventory for Quality of Life in Children and Adolescents.

[39] Becker K.D., Chorpita B.F., Daleiden E.L. (2011). Improvement in Symptoms Versus Functioning: How do our best treatments measure up?. Adm. Policy Ment. Health.

[40] Ballesteros-Urpi A., Slade M., Manley D., Pardo-Hernandez H. (2019). Conceptual framework for personal recovery in mental health among children and adolescents: A systematic review and narrative synthesis protocol. BMJ Open.

[41] Patton G.C., Coffey C., Romaniuk H., Mackinnon A., Carlin J.B., Degenhardt L., Olsson C.A., Moran P. (2014). The prognosis of common mental disorders ion adolescents: A 14-year prospective study. Lancet.

[42] Schneidtinger C., Haslinger-Baumann E. (2019). The lived experience of adolescent user of mental health services in Vienna, Austria: A qualitative study of personal recovery. J. Adolesc. Psychiatr. Nurs..

[43] Bickman L., Nurcombe B., Townsend C., Belle M., Schut J., Karver M. (1998). Consumer Measurement Systems in Child and Adolescent Mental Health.

[44] Kelly S.D., Bickman L. (2009). Beyond outcomes monitoring: Measurement feedback systems in child and adolescent practice. Curr. Opin. Psychiatry.

